# HSF1 transcriptional activity mediates alcohol induction of *Vamp2* expression and GABA release

**DOI:** 10.3389/fnint.2013.00089

**Published:** 2013-12-11

**Authors:** Florence P. Varodayan, Neil L. Harrison

**Affiliations:** ^1^Department of Neuroscience, Columbia UniversityNew York City, NY, USA; ^2^Department of Anesthesiology, Columbia UniversityNew York City, NY, USA; ^3^Department of Pharmacology, Columbia UniversityNew York City, NY, USA

**Keywords:** alcohol/ethanol, γ-aminobutyric acid (GABA), heat shock factor 1 (HSF1), soluble *N*-ethylmaleimide-sensitive factor attachment protein receptor (SNARE), synaptobrevin/vesicle-associated membrane protein (VAMP)

## Abstract

Many central synapses are highly sensitive to alcohol, and it is now accepted that short-term alterations in synaptic function may lead to longer-term changes in circuit function. The regulation of postsynaptic receptors by alcohol has been well studied, but the mechanisms underlying the effects of alcohol on the presynaptic terminal are relatively unexplored. To identify a pathway by which alcohol regulates neurotransmitter release, we recently investigated the mechanism by which ethanol induces *Vamp2*, but not *Vamp1*, in mouse primary cortical cultures. These two genes encode isoforms of synaptobrevin, a vesicular soluble N-ethylmaleimide-sensitive factor attachment protein receptor (SNARE) protein required for synaptic vesicle fusion. We found that alcohol activates the transcription factor heat shock factor 1 (HSF1) to induce *Vamp2* expression, while *Vamp1* mRNA levels remain unaffected. As the *Vamp2* gene encodes a SNARE protein, we then investigated whether ethanol exposure and HSF1 transcriptional activity alter neurotransmitter release using electrophysiology. We found that alcohol increased the frequency of γ-aminobutyric acid (GABA)-mediated miniature IPSCs via HSF1, but had no effect on mEPSCs. Overall, these data indicate that alcohol induces HSF1 transcriptional activity to trigger a specific coordinated adaptation in GABAergic presynaptic terminals. This mechanism could explain some of the changes in synaptic function that occur soon after alcohol exposure, and may underlie some of the more enduring effects of chronic alcohol intake on local circuit function.

## Introduction

Alcohol abuse and dependence is a major global health problem, but little is understood about the neuroadaptations that underlie the development of this disease. Considerable evidence suggests that transient molecular changes can occur during a single alcohol exposure, and that these can persist over time, as individual neurons respond to each and every alcohol exposure in a systematic and coordinated manner (Nestler, 2001; Koob, 2006). In particular, many central synapses are highly responsive to alcohol, and alterations in synaptic function may lead to long lasting changes in local circuitry.

While the mechanisms underlying the postsynaptic effects of alcohol on a variety of neurotransmitter receptors are well studied (Lovinger, 1997; Harris, 1999), only in the last decade have researchers begun to investigate the effects of acute and chronic ethanol treatment on neurotransmitter release (Criswell and Breese, 2005; Siggins et al., 2005; Weiner and Valenzuela, 2006). Acute application of ethanol increases γ-aminobutyric acid (GABA) release in the central amygdala (CeA; Roberto et al., 2003), cerebellum (Carta et al., 2004) and ventral tegmental area (VTA; Theile et al., 2008), as revealed by increased miniature inhibitory postsynaptic current (mIPSC) frequency and paired-pulse depression. In addition, mIPSC frequency is increased in the VTA of mice administered a single ethanol dose one day prior to recording (Melis et al., 2002) and in the CeA of chronically ethanol-treated rats (Roberto et al., 2004). Despite these findings that alcohol increases GABA release, the effects of alcohol on synaptic vesicle fusion machinery are not well understood.

Soluble *N*-ethylmaleimide-sensitive factor attachment protein receptors (SNARE) proteins play a critical role in neurotransmitter release. During synaptic vesicle fusion, synaptotagmin 1 binds to the vesicular SNARE (v-SNARE) synaptobrevin/vesicle-associated membrane protein (VAMP) and plasma membrane phospholipids (Martens et al., 2007). This pulls the two membranes into closer proximity and promotes zippering of synaptobrevin and plasma membrane target SNAREs (t-SNAREs: SNAP-25, syntaxin-1), triggering vesicle fusion and neurotransmitter release. We have found that a subset of genes encoding SNAREs and SNARE-associated proteins are induced by acute alcohol exposure, including synaptotagmin 1 (*Syt1*), *Vamp2*, and *Snap25* (Varodayan et al., 2011).

In particular, our laboratory showed that alcohol exposure rapidly induced *Vamp2* gene expression, but not *Vamp1* (Varodayan et al., 2011). These two genes encode distinct isoforms of synaptobrevin, but are not strictly redundant as VAMP2-deficient mice die shortly after birth (Schoch et al., 2001) and mice with a VAMP1 null mutation develop a neuromuscular wasting disease and die within 2 weeks (Nystuen et al., 2007). It is possible that these outcomes are linked to differential patterns of *Vamp* gene expression throughout the body and in particular, the central nervous system. *Vamp2* gene expression is high throughout the rodent forebrain, including across the entire cortex (Gene Expression Nervous System Atlas [GENSAT; Gong et al., 2007] Project. NINDS Contracts N01NS02331 & HHSN271200723701C to The Rockefeller University, New York, NY), whereas *Vamp1* mRNA levels predominate in the diencephalon, midbrain, brainstem, and spinal cord (Trimble et al., 1990; Nystuen et al., 2007). Closer analysis of synaptobrevin expression in the cerebral cortex, however, found that VAMP1 and VAMP2 are co-expressed at different rates in GABAergic and glutamatergic axon terminals, suggesting that there are underlying cell type specific differences in their patterns of expression (Morgenthaler et al., 2003; Bragina et al., 2010).

As synaptobrevin is intimately involved in synaptic vesicle fusion, changes in its expression levels may alter neurotransmitter release. We reasoned that a careful study of the effects of alcohol on *Vamp2* gene expression might reveal a molecular mechanism by which alcohol can alter neurotransmitter release.

## Materials and methods

The Columbia University Institutional Animal Care and Use Committee approved all protocols involving the use of experimental animals in this study.

### Cortical neuronal cell culture and ethanol exposure

Cortical neurons were cultured from mixed gender embryonic day 17–18 C57BL/6 mice (Harlan Laboratories, Indianapolis, IN; Charles River Laboratories, Wilmington, MA) as previously described (Huettner and Baughman, 1986) with modifications (Ma et al., 2004; Varodayan et al., 2011).

Cortical neurons were cultured for 14–21 days *in vitro* (DIV) and then exposed to ethanol (final concentrations 10–150 mM; Sigma-Aldrich, St. Louis, MO) or vehicle Dulbecco's phosphate-buffered saline control (Invitrogen, Carlsbad, CA) for specific time periods (15 min–24 h), by addition directly to the culture medium. All transfection protocols and electrophysiology recordings were performed after 16 DIV.

### Quantitative real-time polymerase chain reaction (qPCR) analyses of mRNA levels

qPCR was carried out as previously described (Ma et al., 2004; Pignataro et al., 2007; Varodayan et al., 2011). Briefly, total RNA was isolated from the neurons using TRIzol (Invitrogen) and cDNA was prepared with the iScript cDNA synthesis kit (Bio-Rad, Hercules, CA). The first-strand reverse transcribed cDNA was then used as a template for PCR amplification with the appropriate specific primer pairs listed below. qPCR reactions were carried out with iQ SYBR Green Supermix (Bio-Rad) using a Chromo4 Real-Time PCR machine (Bio-Rad).

In preliminary experiments, the *Vamp2* cDNA concentration was normalized against *Actb*, *Gapdh* and *18S* [gene encoding ribosomal protein 18S] (QuantumRNA Internal Standards, Ambion, Austin, TX) cDNA within the same sample. As the results were not significantly different among the three internal standards, for all subsequent experiments the cDNA concentration for the gene of interest was normalized against the concentration of *Actb* cDNA within the same sample. The final results were expressed as percentage of increase vs. the control.

The following primers (and acquisition temperatures) were used for qPCR: *Actb* (82°C) forward (5′-TCATGAAGTGTGACGTTGACATCCGT-3′), reverse (5′-CCTAGAAGCATTTGCGGTGCACGATG-3′); *Gapdh* (77°C) forward (5′-AACTTTGGCATTGTGGAAGG-3′), reverse (5′-ACACATTGGGGGTAGGAACA-3′); *Vamp1* (72°C) forward (5′-AGCATCACAATTTGAGAGCAGT-3′), reverse (5′-GATGGCACAGATAGCTCCCAG-3′); *Vamp2* (76°C) forward (5′-GCTGGATGACCGTGCAGAT-3′), reverse (5′-GATGGCGCAGATCACTCCC-3′).

### RNA interference experiments

RNA interference experiments were performed with 20–25 nucleotide small interference RNA (siRNA), as previously described (Pignataro et al., 2007; Varodayan et al., 2011). Briefly, cultured cortical neurons were transfected with *Hsf1* or control scrambled siRNAs (Santa Cruz Biotechnology, Santa Cruz, CA) for 1 h at 37°C. Cells were washed once and the transfection medium was replaced with conditioned medium for another 24 h prior to ethanol or vehicle treatment.

### Constitutively active and inactive heat shock factor 1 (Hsf1) constructs

We made use of a constitutively transcriptionally active form of HSF1 (*Hsf1*-act, BH-S), as well as a dominant negative mutant form of HSF1 that suppresses HSF1 transcriptional activity (*Hsf1*-inact, AV-ST), as previously described (Pignataro et al., 2007; Varodayan et al., 2011). *Hsf1*-act has amino acids 203–315 deleted in the regulatory domain of HSF1 (Zuo et al., 1995), while *Hsf1*-inact has a deletion in the transcription activation domain of amino acids 453–523 (Zuo et al., 1995). Both constructs were generated by Dr. Richard Voellmy (University of Miami) and cloned into pcDNA3.1+ (Invitrogen). Transfections were performed with 1 μ g of DNA and 9 μ L of nupherin (Enzo Life Sciences, Farmingdale, NY), and sister cultures were transfected with the empty pcDNA3.1+ vector as sham controls, as described previously (Pignataro et al., 2007; Varodayan et al., 2011).

### Electrophysiology recordings

Whole-cell voltage clamp patch recordings were used to determine the effects of ethanol on excitatory and inhibitory miniature postsynaptic currents (mPSCs). After ethanol exposure for 5–15 min or 4–8 h, cells were washed once with fresh media to remove ethanol before being incubated in an external solution containing: 124 mM NaCl, 2.5 mM KCl, 2 mM MgSO_4_, 1.25 mM NaH_2_PO_4_, 2 mM CaCl_2_, 26 mM NaHCO_3_ and 10 mM glucose (all Sigma), at 310 mOsm, and pH 7.4. mPSCs were recorded in the presence of tetrodotoxin (TTX; 100 nM; Tocris, Bristol, UK), with excitatory events (mEPSCs) isolated using SR 95531 hydrobromide (gabazine; 20 μM; Tocris) and inhibitory events (mIPSCs) isolated using 2,3-Dioxo-6-nitro-1,2,3,4-tetrahydrobenzo[f]quinoxaline-7-sulfonamide (NBQX; 10 μM; Tocris) and D-(-)-2-Amino-5-phosphonopentanoic acid (D-APV; 30 μM; Tocris). Patch pipettes were pulled on a Flaming/Browning micropipette puller (Sutter Instrument Company, Novato, CA) from thinwall glass (World Precision Instruments, Sarasota, FL) with a resistance of 3–6 MΩ. The pipettes were filled with an internal solution containing: 140 mM CsCl, 4 mM NaCl, 1 mM MgCl_2_, 0.05 mM EGTA, 2 mM ATP-Mg^2+^, 0.3 mM GTP-Na^+^ and 10 mM HEPES (all Sigma), at 290 mOsm, and pH 7.25. It should be noted that using cesium in the internal solution can increase protein kinase A (PKA) activity within the recording neuron (Vargas et al., 1999); however, this effect should be purely postsynaptic and of minor concern in this study. Membrane potentials were clamped at −70 mV and currents were recorded with an Axopatch 200B patch-clamp amplifier (Molecular Devices, Sunnyvale, CA).

Data were acquired with pClamp 10.3 software (Molecular Devices), filtered at 2 kHz and digitized at 20 kHz. Each recording was a minimum of 6 min long, with the final minute of data analyzed to identify mPSCs. The mPSCs were detected using the Mini Analysis Program 6.0.7 (Synaptosoft, Fort Lee, NJ) with threshold criteria of 5 pA. To assess mPSC frequency and kinetics, the recording trace was visually inspected and only the automatically detected events with a stable baseline, sharp rising phase, and single peak were used.

### Statistical analyses

The qPCR data were analyzed by one-way *ANOVA* followed by Dunnett's multiple-comparison *post-hoc* tests. In these experiments, *n* represents the total number of triplicate sample values averaged into each data point, and each data point contains at least three biological replicates. Electrophysiology numerical data were analyzed using a two-tailed unpaired *t*-test or by one-way *ANOVA* followed by Dunnett's multiple comparison *post-hoc* tests. In these experiments, *n* represents the number of cells tested from at least three biological replicates. All data are presented as mean ± s.e.m and the details of the statistical analyses are included in the appropriate figure legends.

## Results

### Alcohol increases *Vamp2* gene expression

**Figure 1:**
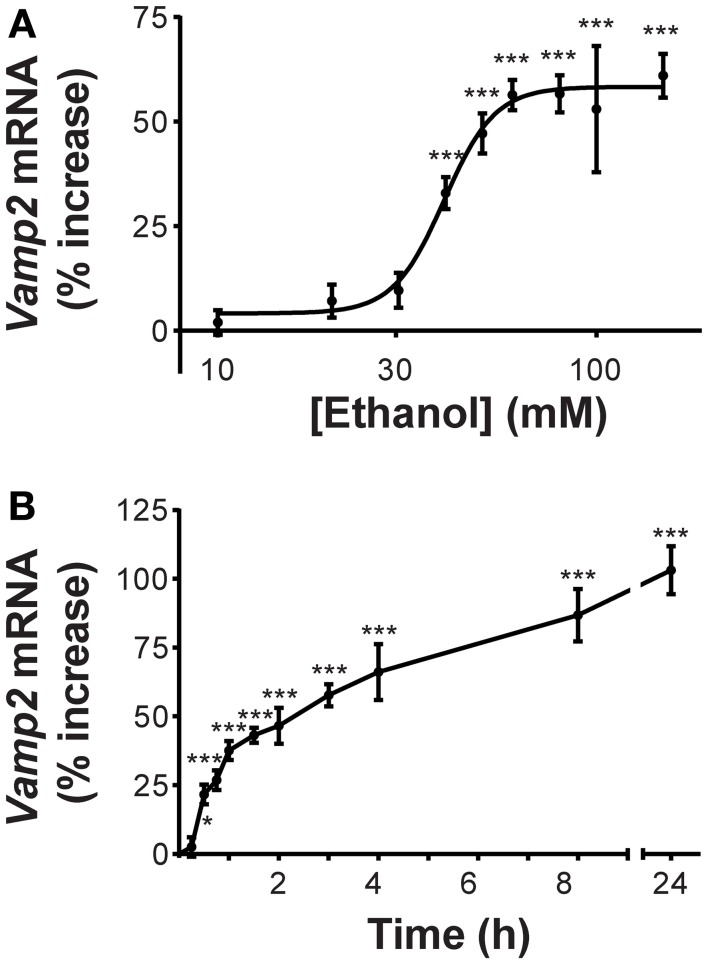
**Ethanol increases *Vamp2* gene expression. (A)** *Vamp2* mRNA levels increase after 1 h treatment with different concentrations of ethanol, as measured by qPCR. The half-maximal activation of *Vamp2* was calculated as 40 ± 6 mM [*n* ≥ 6; *F*_(9, 72)_ = 20.45; *p* < 0.0001]. **(B)** *Vamp2* mRNA levels increase after 60 mM ethanol exposure over time [*n* ≥ 6; *F*_(10, 195)_ = 39.58; *p* < 0.0001; ^*^P < 0.05, ^***^P < 0.001].

Our initial experiments confirmed our previous finding that *Vamp2* is an alcohol-responsive gene (Varodayan et al., 2011). We found that ethanol induction of *Vamp2* mRNA levels was concentration-dependent (Figure 1A), with the *Vamp2* gene responding modestly to ethanol concentrations more relevant to social intoxication (10–30 mM) and strongly to the high ethanol concentrations similar to those measured in blood samples of chronic alcoholics (80–100 mM) (Urso et al., 1981). The ethanol effect on *Vamp2* gene expression showed a half-maximal activation at 40 ± 6 mM (33 ± 4% increase compared with ethanol-naïve control) and saturated at 80 mM (57 ± 5% increase). These brief exposures to high ethanol concentrations were not toxic to the neurons, as treatment with 100 mM ethanol caused little, if any, apoptosis, as previously reported (Pignataro et al., 2007). The time course of the activation of *Vamp2* transcription by 60 mM ethanol was rapid, with *Vamp2* gene expression significantly increased at 30 min of exposure (22 ± 4% increase; Figure 1B). *Vamp2* mRNA levels continued to rise during 8 h of 60 mM ethanol exposure (87 ± 10% increase) and were further increased at 24 h of continuous exposure (103 ± 9% increase).

### HSF1 transcriptional activation mediates alcohol induction of *Vamp2* gene expression

**Figure 2:**
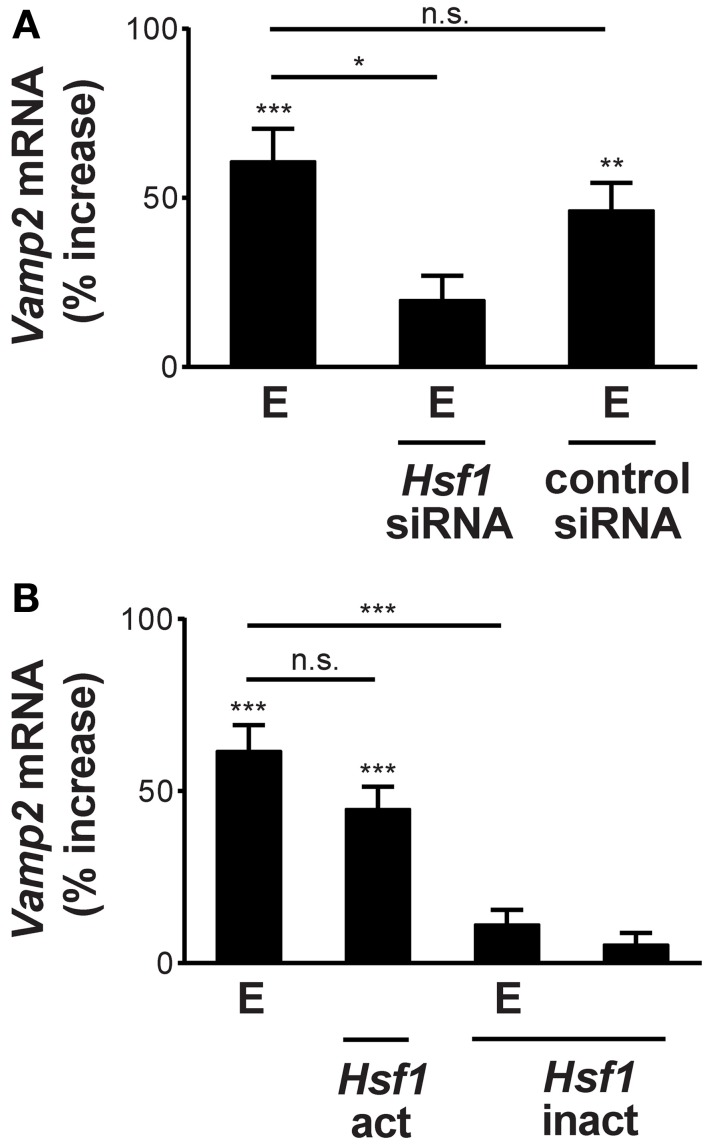
**Ethanol induction of the *Vamp2* gene requires transcriptionally activated HSF1. (A)** HSF1 knock-down inhibits *Vamp2* gene induction by ethanol. Overnight pretreatment of neurons with *Hsf1* siRNA reduced the effects of 60 mM ethanol exposure for 1 h (E) on *Vamp2* mRNA levels, while pretreatment with control siRNA had no effect [*n* ≥ 6; *F*_(3, 44)_ = 13.55; *p* < 0.001]. **(B)** Stimulation of the *Vamp2* gene by ethanol is mediated by transcriptionally activated HSF1. Cortical neurons transfected with *Hsf1*-act showed increased *Vamp2* mRNA expression, similar to the gene's induction by 60 mM ethanol for 1 h (E). *Hsf1*-inact transfection reduced ethanol induction of the *Vamp2* gene, while *Hsf1*-inact transfection alone had no effect on basal *Vamp2* mRNA levels. Control cultures were sham transfected with an empty pcDNA3.1+ construct [*n* ≥ 6; *F*_(4, 73)_ = 27.53; *p* < 0.001; ^*^P < 0.05, ^**^P < 0.01, ^***^P < 0.001, or n.s. denotes no significance].

A subset of alcohol-responsive genes are known to be up-regulated via activation of the transcription factor, heat shock factor 1 (HSF1; Pignataro et al., 2007, 2013; Varodayan et al., 2011). To investigate whether HSF1 mediates *Vamp2* gene induction by ethanol, we altered HSF1 protein expression and assessed changes in *Vamp2* mRNA levels after ethanol treatment. We found that knock-down of HSF1 protein, using neuronal transfection with *Hsf1* siRNA, decreased *Vamp2* gene induction after ethanol exposure (from 61 ± 10% increase to 20 ± 7%; Figure 2A). Transfection with control siRNA had no effect on basal *Vamp2* mRNA levels (Figure 2A).

Previous work from our laboratory demonstrated that the *Vamp1* gene was not induced when primary cortical culture was exposed to 60 mM ethanol for 1 h (Varodayan et al., 2011). Here we found that the knock-down of HSF1 protein, using neuronal transfection of *Hsf1* siRNA, had no effect on *Vamp1* mRNA levels.

To confirm the role of HSF1 in mediating *Vamp2* gene induction, we used a constitutively active *Hsf1* construct (*Hsf1*-act). This construct encodes a transcriptionally active HSF1 protein that can directly induce heat shock protein (*Hsp*) gene transcription in the absence of heat stress (Zuo et al., 1995; Xia et al., 1999). Neuronal transfection of this construct increased *Vamp2* gene expression to a level similar to that seen after 1 h of 60 mM ethanol exposure (42 ± 6% increase; Figure 2B). Conversely, a dominant-negative *Hsf1* construct (*Hsf1*-inact), which encodes a transcriptionally inactive HSF1 protein that suppresses stress-induced *Hsp* gene expression (Zuo et al., 1995; Xia et al., 1999), abolished the effect of ethanol exposure on *Vamp2* mRNA levels (from a 62 ± 7% increase to 11 ± 4%; Figure 2B). *Hsf1*-inact transfection alone had no effect on basal *Vamp2* gene expression (Figure 2B). These experiments reveal that HSF1 transcriptional activity stimulates *Vamp2* mRNA levels and mediates ethanol induction of the *Vamp2* gene. In the case of the *Vamp1* gene, altering HSF1 transcriptional activity by neuronal transfection with either *Hsf1*-act or *Hsf1*-inact and ethanol treatment had no effect on mRNA levels.

### Alcohol increases mIPSC frequency

As *Vamp2* is one of several alcohol-responsive genes that encode proteins intimately involved in synaptic vesicle fusion (Varodayan et al., 2011), we explored whether ethanol alters neurotransmitter release. To investigate this potential mechanism, we used whole-cell voltage clamp electrophysiology to record mPSCs in ethanol exposed cultured cortical neurons treated with 100 nM TTX to block action potential-dependent neurotransmitter release. In these experiments, increased mPSC frequency indicates alterations in the presynaptic terminal leading to an increased probability of synaptic vesicle fusion and neurotransmitter release, while increased mPSC amplitude reflects an increase in postsynaptic receptor sensitivity to the released neurotransmitter, possibly due to changes in receptor subunit composition or the number of receptors present (De Koninck and Mody, 1994; Otis et al., 1994).

**Figure 3:**
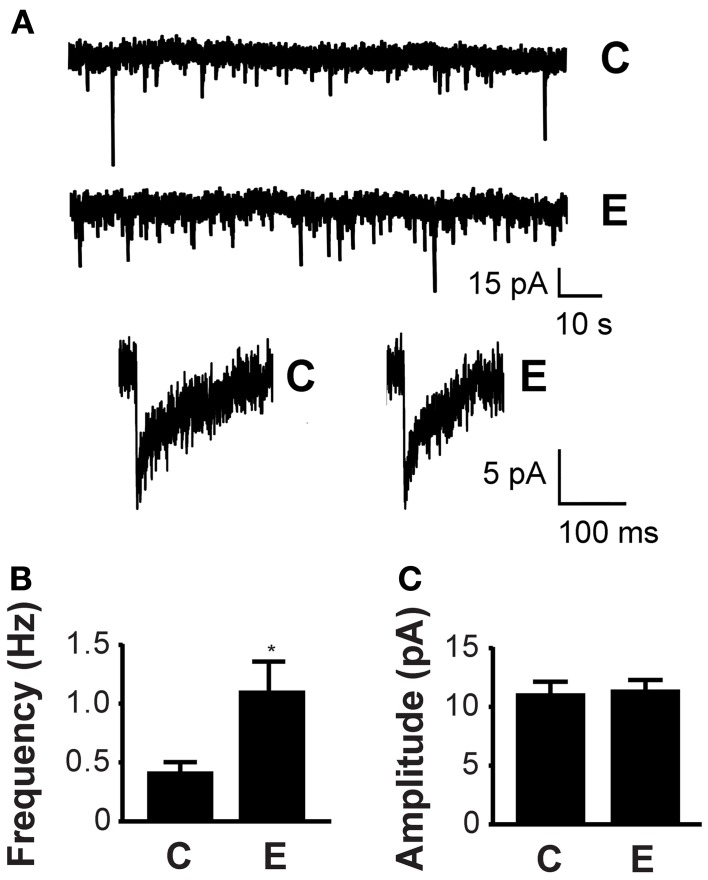
**Ethanol increases mIPSC frequency. (A)** Whole-cell voltage clamp electrophysiology recordings demonstrate that ethanol increases the probability of GABA release. The representative current traces in the upper panel were recorded in neurons exposed to 60 mM ethanol for 4–8 h (E) or a vehicle control (C). Magnified representative mIPSC events from control and ethanol-treated neurons are shown in the lower panel. **(B)** Ethanol increases the mean frequency of mIPSCs in neurons treated with ethanol (E) compared to vehicle control [C; *n_C_* = 19, *n_E_* = 22; *t*_(39)_ = 2.51; *p* < 0.05]. **(C)** Ethanol does not alter the mean amplitude of mIPSCs in neurons exposed to ethanol (E) or vehicle control [C; *n_C_* = 19, *n_E_* = 22; *t*_(39)_ = 0.24; *p* = 0.81; ^*^P < 0.05].

We first evaluated the effects of 60 mM ethanol exposure for 4–8 h on inhibitory currents (mIPSCs) by recording in the presence of 30 μM D-APV and 10 μM NBQX to block glutamatergic events. Notably, we found that ethanol increased the frequency of mIPSCs compared to control neurons, as seen in the representative traces and bar graph (*f_C_* = 0.42 ± 0.08 Hz, *f_E_* = 1.11 ± 0.23 Hz; Figure 3A upper panel, **B**). Ethanol had no effect on mIPSC amplitude (*A_C_* = 10.68 ± 0.93 pA, *A_E_* = 10.98 ± 0.74 pA; Figure 3A lower panel, **C**) or the rise time constant (*t*_*rC*_ = 3.21 ± 0.22 ms, *t*_*rE*_ = 3.24 ± 0.16 ms), but shortened the decay time constant (*t*_*dC*_ = 12.59 ± 2.05 ms, *t*_*dE*_ = 8.19 ± 0.78 ms; Table 1). The mIPSCs were totally blocked by the perfusion of 20 μM gabazine and partially recovered upon washout in all 5 cells tested, indicating that these events are GABAergic. Similar experiments conducted after 5–15 min of 60 mM ethanol exposure revealed no change in mIPSC frequency (*f_C_* = 0.47 ± 0.08 Hz, *f_E_* = 0.55 ± 0.13 Hz; *n_C_* = 13, *n_E_* = 17) or amplitude (*A_C_* = 9.40 ± 0.95 pA, *A_E_* = 8.03 ± 0.78 pA; *n_C_* = 13, *n_E_* = 17), suggesting that this mechanism of ethanol-induced GABA release may require the prolonged processes of transcription and translation.

**Table 1:** **A single ethanol exposure alters mPSC kinetics**.

	**Treatment**	**Frequency (Hz)**	**Amplitude (pA)**	**Rise time (ms)**	**Decay time (ms)**
mIPSC	C (*n* = 22)	0.42 ± 0.08	10.68 ± 0.93	3.21 ± 0.22	12.59 ± 2.05
	E (*n* = 19)	1.11 ± 0.23^*^	10.98 ± 0.74	3.24 ± 0.16	8.19 ± 0.78^*^
mEPSC	C (*n* = 22)	0.44 ± 0.13	6.10 ± 0.13	1.94 ± 0.16	0.79 ± 0.15
	E (*n* = 22)	0.40 ± 0.07	6.79 ± 0.38	2.67 ± 0.22^*^	1.02 ± 0.15

* P < 0.05.

Data are obtained from neurons exposed to 60 mM ethanol for 4–8 h and control neurons.

To study the effects of ethanol on excitatory currents (mEPSCs), we used 20 μM gabazine to block GABA_A_ receptor-mediated events. We found no change in mEPSC frequency (*f_C_* = 0.44 ± 0.13 Hz, *f_E_* = 0.40 ± 0.07 Hz; *n_C_* = 22, *n_E_* = 22) or amplitude (*A_C_* = 6.10 ± 0.13 pA, *A_E_* = 6.79 ± 0.38 pA; *n_C_* = 22, *n_E_* = 22) after 60 mM ethanol exposure for 4–8 h. Details of mEPSC kinetics are displayed in Table 1.

### HSF1 transcriptional activity mediates alcohol induction of mIPSC frequency

**Figure 4:**
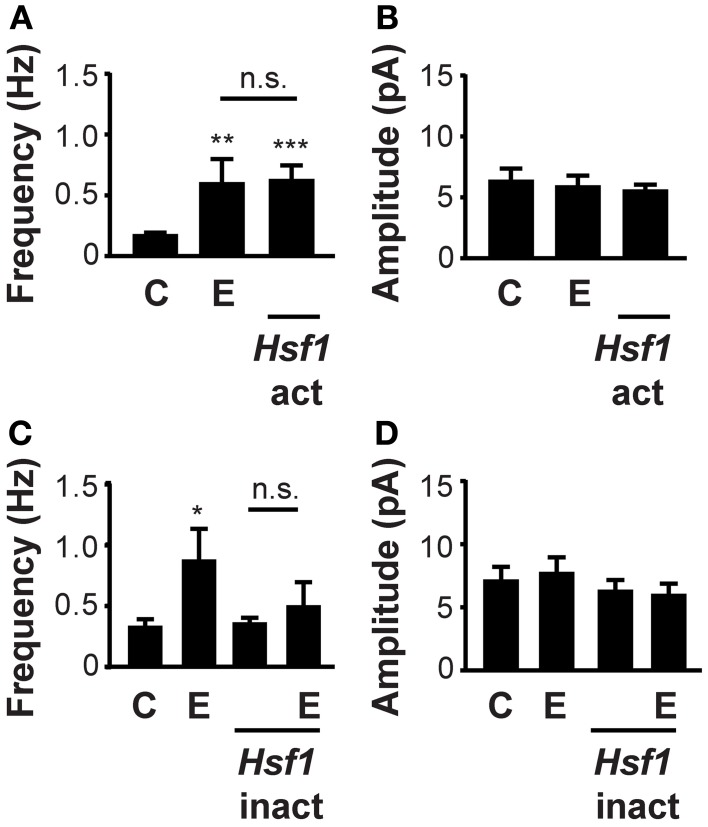
**Increased GABA release after ethanol exposure requires HSF1 transcriptional activity. (A)** HSF1 transcriptional activity increases the probability of GABA release. *Hsf1*-act transfection increased mIPSC frequency, similar to the level seen with 60 mM ethanol exposure for 4–8 h (E). Control cultures were sham transfected with an empty pcDNA3.1+ construct [*C*; *n_C_* = 15, *n_E_* = 17, *n_Hsf_*_1_*_act_* = 19; *F*_(2, 48)_ = 3.46; *p* < 0.05]. **(B)** HSF1 activity does not alter the mean mIPSC amplitude in neurons transfected with an *Hsf1*-act construct, exposed to ethanol (E) or control sham transfected [*C*; *n_C_* = 15, *n_E_* = 17, *n_Hsf_*_1_*_act_* = 19; *F*_(2, 48)_ = 0.32; *p* = 0.73]. **(C)** Ethanol stimulation of mIPSC frequency is mediated by activated HSF1. *Hsf1*-inact transfection reduced the effects of ethanol (E) on mIPSC frequency. *Hsf1*-inact transfection alone had no effect on mIPSC frequency compared to control cultures sham transfected with empty pcDNA3.1+ construct [*C*; *n_C_* = 16, *n_E_* = 10, *n_Hsf_*_1_*_inact_* = 12, *n_Hsf_*_1_*_inact_*_+*E*_ = 14; *F*_(3, 48)_ = 2.56; *p* = 0.07]. **(D)** HSF1 activity does not alter the mean amplitude of mIPSCs in neurons transfected with an *Hsf1*-inact construct, exposed to ethanol (E) or vehicle control [*C*; *n_C_* = 16, *n_E_* = 10, *n_Hsf_*_1_*_inact_* = 12, *n_Hsf_*_1_*inact*_+*E*_ = 14; *F*_(3, 48)_ = 0.0639; *p* = 0.60; ^*^P < 0.05, ^**^P < 0.01, ^***^P < 0.001, or n.s. denotes no significance].

To investigate whether HSF1 transcriptional activity mediates the increased mIPSC frequency observed after ethanol exposure, we altered HSF1 protein expression and assessed mIPSC kinetics. Neuronal transfection of *Hsf1*-act increased mIPSC frequency similar to the frequency observed after ethanol exposure (*f_C_* = 0.18 ± 0.01 Hz, *f_E_* = 0.61 ± 0.19 Hz, *f_Hsf_*_1_*_act_* = 0.63 ± 0.11 Hz; Figure 4A). Conversely, the dominant-negative *Hsf1*-inact construct abolished the effect of ethanol exposure on mIPSC frequency (*f_C_* = 0.34 ± 0.05 Hz, *f_E_* = 0.88 ± 0.25 Hz, *f_Hsf_*_1_*_inact_* = 0.37 ± 0.04 Hz, *f_Hsf_*_1_*_inact_*_+*E*_ = 0.51 ± 0.19 Hz), while *Hsf1*-inact transfection alone had no effect on mIPSC frequency (Figure 4C). No changes were observed in amplitudes (Figures 4B,D), rise times or decay times after transfection with either the *Hsf1*-act or *Hsf1*-inact constructs. These experiments reveal that HSF1 transcriptional activity increases GABA release and mediates ethanol induction of mIPSC frequency. In summary, in this study we have shown that ethanol acts via HSF1 to increase the gene expression of a specific subset of proteins involved in synaptic vesicle fusion and stimulate GABA release.

## Discussion

Ethanol alters GABA release throughout the central nervous system (Criswell and Breese, 2005; Siggins et al., 2005; Weiner and Valenzuela, 2006), but the underlying mechanisms are largely unknown. We recently showed that a subset of genes encoding SNARE complex proteins is induced by alcohol exposure. In particular, we found that alcohol differentially regulates two genes encoding synaptobrevin isoforms, rapidly inducing the *Vamp2* gene, but not *Vamp1*, and were therefore interested in the mechanism underlying this difference (Varodayan et al., 2011). Here, we show that HSF1 transcriptional activity mediates ethanol induction of *Vamp2* gene expression in cortical neurons. Since VAMP2 is intimately involved in synaptic vesicle fusion, we then investigated whether alcohol acts via HSF1 to alter neurotransmitter release. We found that HSF1 transcriptional activity mediates ethanol-induced GABA release, but has no effect on glutamatergic synaptic vesicle fusion.

### A single alcohol exposure induces SNARE gene expression

We have previously shown that acute alcohol exposure rapidly induces transcription of some SNARE complex proteins, including the *Vamp2*, *Syt1* and *Snap25* genes, but not the *Vamp1*, *Stx1a*, and *Syp* genes (Varodayan et al., 2011). In this study we investigated the mechanism underlying *Vamp2* gene induction by alcohol. There are few, if any, comparable studies on the effects of alcohol on *Vamp2* gene expression. Interestingly, a recent transcriptome profiling study used tissue from alcoholic human brain cortices to identify *Vamp2* as a hub gene that is likely to have high functional significance in biological processes associated with alcohol dependence (Ponomarev et al., 2012).

### A molecular mechanism underlying the effects of a single alcohol exposure on SNARE gene expression

We found that ethanol induction of the *Vamp2* gene is mediated by HSF1 activity. Transcriptional activation of HSF1 is a multistep process that involves: HSF1 translocation from the cytoplasm, where it is sequestered by chaperone proteins, to the nucleus; HSF1 trimerization and inducible hyperphosphorylation; and HSF1 binding to a DNA element to stimulate transcription (Cotto et al., 1997). We have previously shown that 60 mM ethanol exposure of primary cortical culture induces HSF1 translocation into the nucleus (Pignataro et al., 2007), phosphorylates HSF1(Varodayan et al., 2011) and stimulates *Hsp* gene expression (Pignataro et al., 2007), indicating that ethanol promotes HSF1 transcriptional activity. Several other laboratories have also reported an association between alcohol exposure and HSF1-dependent gene induction, including microarray studies where alcohol treatment increased *Hsp* gene expression (Lewohl et al., 2000; Gutala et al., 2004; Worst et al., 2005). In addition, we have previously reported that ethanol acts via HSF1 to induce the *Syt1* gene and the gene encoding the α4 subunit of the GABA_A_ receptor (Pignataro et al., 2007; Varodayan et al., 2011). As a whole, our current studies strongly suggest that HSF1 transcriptional activity mediates the effects of alcohol on a subset of alcohol-responsive genes, including some SNARE proteins. As the SNARE proteins are intimately involved in synaptic vesicle fusion, this raises the interesting question of whether the neuronal response to alcohol includes alterations in neurotransmitter release.

### A single alcohol exposure causes a wave of transient presynaptic adaptations leading to changes in GABA release

Changes in GABA release after ethanol exposure have been reported in the last decade (Criswell and Breese, 2005; Siggins et al., 2005; Weiner and Valenzuela, 2006). We found that mIPSC frequency increased in cortical neurons exposed to 60 mM ethanol for 4–8 h, but not 5–15 min, suggesting that this mechanism of ethanol-induced GABA release may require the prolonged processes of transcription and translation. Similar experiments by the Morrow laboratory found an unchanged mIPSC frequency in cultured cortical rat neurons exposed to 50 mM ethanol for either 4 h or 1–7 days (Fleming et al., 2009; Werner et al., 2011). As a whole, these results suggest that the increase in mIPSC frequency after a single ethanol exposure may be a transient neuronal adaptation. Studies conducted *in vivo* also showed changes in mIPSC frequency across the rodent brain, with Melis et al. (2002) observing an increase in mIPSC frequency in the VTA of mice injected intraperitoneally with ethanol one day prior to recording. Chronic ethanol-treated rats showed a similar increase in mIPSC frequency in the CeA and this frequency was further increased by the bath application of ethanol, indicating that the acute, and chronic effects of ethanol on GABA release are differentially mediated (Roberto et al., 2004). Overall, these data define a model of transient presynaptic adaptation, where ethanol promotes HSF1 transcriptional activity to induce a temporary increase in GABA release. This transient change in neurotransmitter release may lead to more permanent synaptic modifications, especially as the cycle is repeated with multiple exposures to alcohol.

### A molecular mechanism underlying some of the effects of a single alcohol exposure on GABA release

The mechanisms underlying the effects of ethanol exposure on GABA release have been largely unstudied. Our detailed analysis revealed that ethanol treatment of cultured cortical neurons increases GABA release via HSF1 transcriptional activity, although it is likely that a variety of alternate and overlapping mechanisms underlie the similar changes observed after different ethanol exposure models and across brain regions. For example, ethanol application in the cerebellum rapidly increases the number of mIPSC events in interneurons via activation of both AC/PKA and PLC/PKC pathways and internal calcium store release (Kelm et al., 2007, 2008, 2010). The effects of alcohol administration on these kinase pathways provide for a relatively fast GABAergic neuronal response, while the enhanced GABA release that occurs after chronic ethanol exposure is likely to be regulated by longer-lasting changes in gene expression that are triggered by HSF1 and other transcription factors.

### A single alcohol exposure causes a wave of transient postsynaptic adaptations leading to changes in GABA receptor sensitivity

The synapse is a highly responsive structure and perturbations in presynaptic activity are typically met with an adaptive postsynaptic response, and vice versa. We found that treatment of cortical neurons with ethanol for 4–8 h shortened mIPSC decay time, an indication of changes in postsynaptic GABA_A_ receptor subunit composition or number. mIPSC decay time also decreased in cultured rat cortical neurons exposed to ethanol for 4 h and 1 day, and recovered after 2–7 days (Fleming et al., 2009; Werner et al., 2011). A similar decrease in mIPSC decay time was observed in hippocampal neurons of rats administered a single dose of ethanol and withdrawn 12 h to 7 days, with recovery by day 14 (Liang et al., 2007). Liang et al. (2007) found that these changes in mIPSC kinetics coincided with changes in the surface expression of GABA_A_ receptor subunits. In particular, an increase in α4 expression could cause α4β γ2 GABA_A_ receptors to “crowd” α1β γ2 GABA_A_ receptors out of the synapse, leading to changes in GABA_A_ receptor sensitivity to ethanol. We previously found increased α4 expression in cultured cortical neurons exposed to 60 mM ethanol for 4–8 h (Pignataro et al., 2007), indicating that similar changes in GABA_A_ receptor subunit composition and sensitivity may be occurring in our current study. Overall these data define a model of postsynaptic adaptation to a single dose of ethanol in which there may be a temporary increase in the expression of α4-containing GABA_A_ receptors. This transient change in subunit composition could lead to more permanent synaptic modifications, especially as the cycle is repeated with multiple exposures to alcohol.

### Multiple ethanol exposures could lead to persistent adaptation at the GABA synapse

The data presented here show that a single ethanol exposure induces *Vamp2* gene expression and stimulates GABA release via HSF1 transcriptional activity. Repeated ethanol exposure could result in a persistent adaptation at the GABAergic synapse and lead to enduring changes in the local circuitry that may play a role in the development of alcohol abuse and dependence. It is interesting to note that ethanol's effects on HSF1 appear to alter neurotransmitter release in GABAergic, and not glutamatergic, neurons, and the apparent specificity of this effect among a variety of synapses merits further study.

## Author contributions

Participated in research design: Florence P. Varodayan and Neil L. Harrison. Conducted experiments: Florence P. Varodayan. Performed data analysis: Florence P. Varodayan. Wrote or contributed to the writing of the manuscript: Florence P. Varodayan and Neil L. Harrison.

## Conflict of interest statement

The authors declare that the research was conducted in the absence of any commercial or financial relationships that could be construed as a potential conflict of interest.

## Abbreviations

*ANOVA*: analysis of variance
CRF: corticotrophin-releasing factor
DIV: days *in vitro*
GABA: γ-aminobutyric acid
*Hsf1*: HSF1, heat shock factor 1
*Hsp*: HSP, heat shock protein
mPSC: miniature postsynaptic current
mEPSC: miniature excitatory postsynaptic current
mIPSC: miniature inhibitory postsynaptic current
qPCR: quantitative polymerase chain reaction
SEM: standard error of the mean
siRNA: small interfering RNA
SNAP-25: *Snap-25*, synaptosomal-associated protein 25
SNARE: soluble *N*-ethylmaleimide-sensitive factor attachment protein receptor
*Stx1*: syntaxin-1
*Syt1*: synaptotagmin 1
*Syp1*: synaptophysin 1
TTX: tetrodotoxin
VAMP: *Vamp*, synaptobrevin/vesicle-associated membrane protein
VTA: ventral tegmental area.
